# Cytokine Biosignature of Active and Latent Mycobacterium Tuberculosis Infection in Children

**DOI:** 10.3390/pathogens10050517

**Published:** 2021-04-24

**Authors:** Magdalena Druszczynska, Michal Seweryn, Sebastian Wawrocki, Magdalena Kowalewska-Pietrzak, Anna Pankowska, Wieslawa Rudnicka

**Affiliations:** 1 Department of Immunology and Infectious Biology, Institute of Microbiology, Biotechnology and Im-munology, Faculty of Biology and Environmental Protection, University of Lodz, Banacha 12/16, 90-237 Lodz, Poland; sebastian.wawrocki@gmail.com (S.W.); wieslawa.rudnicka@biol.uni.lodz.pl (W.R.); 2 Biobank Lab, Department of Molecular Biophysics, Faculty of Biology and Environmental Protection, University of Lodz, Banacha 12/16, 90-237 Lodz, Poland; michal.seweryn@biol.uni.lodz.pl; 3 Regional Specialized Hospital of Tuberculosis, Lung Diseases and Rehabilitation in Lodz, Okolna 181, 91-520 Lodz, Poland; magda.kp@interia.pl (M.K.-P.); dr.pankowska@gmail.com (A.P.)

**Keywords:** tuberculosis, latent Mycobacterium tuberculosis infection, cytokines, diagnostics

## Abstract

None of the currently used diagnostic tools are efficient enough in diagnosing Mycobacterium tuberculosis (M.tb) infection in children. The study was aimed to identify cytokine biosignatures characterizing active and latent tuberculosis (TB) in children. Using a multiplex bead-based technology, we analyzed the levels of 53 Th17-related cytokines and inflammatory mediators in sera from 216 BCG-vaccinated children diagnosed with active TB (TB) or latent TB (LTBI) as well as uninfected controls (HC). Children with active TB, compared to HC children, showed reduced serum levels of IL-17A, MMP-2, OPN, PTX-3, and markedly elevated concentrations of APRIL/TNFSF13. IL-21, sCD40L, MMP-2, and IL-8 were significantly differentially expressed in the comparisons between groups: (1) HC versus TB and LTBI (jointly), and (2) TB versus LTBI. The panel consisting of APRIL/TNFSF13, sCD30/TNFRSF8, IFN-α2, IFN-γ, IL-2, sIL-6Rα, IL-8, IL-11, IL-29/IFN-λ1, LIGHT/TNFSF14, MMP-1, MMP-2, MMP-3, osteocalcin, osteopontin, TSLP, and TWEAK/TNFSF12 possessed a discriminatory potential for the differentiation between TB and LTBI children. Serum-based host biosignatures carry the potential to aid the diagnosis of childhood M.tb infections. The proposed panels of markers allow distinguishing not only children infected with M.tb from uninfected individuals but also children with active TB from those with latent TB.

## 1. Introduction

Tuberculosis (TB) caused by *Mycobacterium tuberculosis* (M.tb) remains a global health emergency with 10 million new cases and 1.5 million deaths annually. As estimated, almost 30% of the world population is latently infected with *M.tb*, among whom in approximately 5–10% active TB disease develops. Children belong to the group of individuals highly susceptible to TB. Epidemiological data show that the risk of developing active TB in children ranges from 20% to 40% and is the highest among children below 5 years of age.

TB still remains a serious diagnostic challenge and many pediatric TB cases remain undetected. None of the currently available microbiological, serologic, or molecular tests designed for the diagnosis of active TB are good enough for clinical use in children. Collecting an adequate sample for microbiological diagnosis presents difficulties, particularly for small children, who cannot produce a good sputum specimen. Mycobacterial culture, the gold standard for the confirmation of TB in adults, often fails due to the paucibacillary nature of the disease. Although serum-based antibody assays offer advantages of easy specimen collection and rapidity, none of the serologic tests are sensitive and specific enough for clinical use. Molecular methods (i.e., panels of molecular markers) showed variable sensitivity and specificity in different studies. Therefore, in low TB incidence countries, the diagnosis of pediatric TB is based on non-specific clinical symptoms, the presence of suggestive abnormal radiological features, a positive tuberculin skin test (TST) result, and the history of *M.tb* exposure [1]. These criteria have a limited application in TB endemic regions, because most individuals become TST-positive during childhood or adolescence. According to the current WHO guidelines, all children under 5 years of age who were in close contact with an infectious TB case should be actively screened for infection, and after the exclusion of active disease, they should receive preventive chemotherapy. The implementation of chemoprophylaxis to a child with active TB is a serious error, which leads to failure of the treatment and an increased risk of the emergence of drug-resistant strains [1].

The diagnosis of latent *M. tb* infection (LTBI) in pediatric patients may also be difficult. Two currently available methods for diagnosis of LTBI include the tuberculin skin test (TST) and the interferon-gamma (IFN-γ) release assay (IGRA). The TST, which measures the delayed-type hypersensitivity (DTH) to intradermally injected purified protein derivative (PPD) of *M. tb*, is characterized by variable sensitivity and specificity depending on the cut-off value used. The method was found to have poor specificity and accuracy due to the cross-reactivity with antigens present in other mycobacterial species, including the *M. bovis* Bacillus Calmette–Guerin (BCG) vaccine strain. Moreover, there is accumulating evidence that human genetics play an important role in the development of DTH to PPD [2,3,4]. Interferon-γ release assays, which measure the release of IFN-γ in whole blood samples following in vitro stimulation with *M.tb*-specific antigens, i.e., early secreted antigenic target-6 (ESAT-6), culture filtrate protein 10 (CFP-10), and TB7.7, are recommended as an alternative to the TST. The overall agreement between the IGRA and the TST was found to be between 55% and 95%, depending on age and previous BCG vaccination [5,6,7]. Although the IGRA has higher specificity than the TST and correlates better with the intensity of contact with infectious TB in children, neither of the tests is able to differentiate between active and latent TB [8]. Interestingly, various reports suggest the limited usefulness of the IGRA in children under 5 years of age due to common indeterminate responses [9]. The consequences of missed diagnosis are severe, as untreated children have a high probability of developing active TB, usually within two years after the infection or immediately thereafter. There is a high diversity of the clinical presentation of TB making it difficult to pose the correct diagnosis [10]. At the same time, there is a false expectation and practice to accurately extrapolate the diagnostic tests designed for adults to children. This is caused by the lack of a deep understanding of differences in both the clinical symptoms and the general course of the disease between these two age groups. Hence, there is a need to create new, fast techniques focused on childhood TB.

A great facilitation in diagnostics may be the development of a panel of measurable biological indicators called biomarkers, whose presence or specific concentration indicates the body’s physiological condition, ongoing disease process, or response to implemented treatment [11]. The need for TB biomarkers arises, in part, from the difficulties with TB diagnosis in children; therefore, the analysis of biomarkers seems to be a convenient, sensitive, and non-invasive method of diagnosis. The commonly used biomarkers in adult TB include, among others, transcriptional and metabolic patterns, elements of the mycobacterial cell wall or secretory proteins of mycobacteria, as well as cytokines, chemokines, and other mediators of inflammatory processes. 

The aim of the present study was to ascertain whether blood-based host biosignatures possessing a potential in the diagnosis of adult TB could be used in childhood TB. Using a multiplex cytokine bead-based technology, we analyzed the levels of 53 Th17-related cytokines and inflammatory mediators in sera from 216 BCG-vaccinated Polish children diagnosed with active TB or latent TB as well as uninfected controls to identify biosignatures characterizing childhood active and latent *M.tb* infections.

## 2. Results

### 2.1. Basic Characteristics of the Study Group 

The baseline demographic information for children included in the study is shown in Table 1. The clinical cohort comprised 216 children divided into 3 groups: children with active TB (TB; n = 15) and healthy children with no signs or symptoms of any pulmonary diseases, with latent *M.tb* infection (LTBI; n = 50) or excluded latent *M.tb* infection (HC; n = 151). All children were HIV-negative and BCG-vaccinated as infants, in accordance with the national vaccination program. None of the individuals had evidence of being treated with steroids or other immunosuppressive or anti-tubercular drugs at the time of blood sampling.

There were no differences between the studied groups regarding the sex or BCG vaccination rates. The median age of the TB children (Me 15, IQR (11, 16)) was significantly higher than that of the LTBI (Me 8; IQR (5, 12)) or HC (Me 7; IQR (3, 11)) groups (*p* < 0.05). In all TB patients, Mycobacteria Growth Indicator Tube (MGIT) cultures were positive and speciated for *M.tb*. A positive QFT result was observed in 80% of TB patients, all LTBI individuals, and none of the HC children. TST was positive in 87%, 88%, and 12% of TB, LTBI, and HC subjects, respectively. There were no differences in the median values of WBC, RBC, PLT counts and other hematological parameters between the studied groups (Table 1). The bilirubin, ALT and AST levels did not differ significantly between the groups, but the median CRP concentration was significantly higher in the TB patients than in the LTBI or HC groups (*p* < 0.05) (Table 1).

### 2.2. Identification of Differentially Expressed Th17-Related Cytokines and Inflammatory Mediators in the Study Groups

We compared the median concentrations of studied cytokines and chemokines in the sera from the TB, LTBI and HC children (Table S1). The median concentrations of IL-17A in the sera from the TB and LTBI participants were significantly lower than in the HC individuals. In contrast, the concentrations of IL-1β and IL-23 differed depending on the stage of *M.tb* infection. The levels of these two cytokines were significantly lower in the LTBI than in the HC individuals. However, the concentration of IL-1β and IL-23 in the sera from the TB children did not differ significantly from that detected in the sera from the HC children. The median levels of MMP-2, OPN and PTX-3 were significantly lower in the sera from the TB children compared to the LTBI or HC groups, whereas the concentration of APRIL/TNFSF13 was significantly higher in the TB group in comparison with the LTBI or HC individuals.

In the first linear model-based comparison, we evaluated the differences between the concentrations of proteins in the sera of (1) HC versus TB and LTBI (jointly) and (2) TB versus LTBI. With the aim of significance testing, we utilized an ANOVA approach with 2df. We found that the levels of IL-21 and sCD40L were significantly higher (false discovery rate (FDR) <0.05) in the infected individuals (jointly TB and LTBI) than in the HC group. At the same time, the concentrations of IL-21 and sCD40L were significantly higher in the TB than in the LTBI individuals. In the second linear model-based comparison, we tested all three pairwise differences between the study groups (and applied an ANOVA based 3df approach for testing). As above, we concluded that only IL-21 and sCD40L remained significantly differentially expressed under FDR of 0.05. The results of linear models are presented in Table 2 and Table S2. Subsequently, we conducted identical analyses for the corresponding panel of inflammatory mediators. We concluded that the MMP-2 and IL-8 remained significant in each of the two comparisons—with (1) the MMP-2 having highest concentration in the HC and the lowest in the TB group and (2) the IL-8 having lower expression in the HC and the highest in the TB individuals. The results are summarized in Table 2 and Table S2.

### 2.3. Discriminative Biomarker Potential by Receiver Operating Characteristic (ROC) Analysis

The ROC analysis was performed to indicate the proteins with the expression levels most discriminative for the TB, LTBI, and HC groups. The multiclass AUC was calculated as proposed by Hand et al. with the aid of the pROC package in R [12]. Among the proteins, MMP-2 showed the highest area under the ROC curve (AUC), namely, 0.758 in a 3-group comparison and 0.848, 0.721 and 0.701 for HC versus TB, HC versus LTBI and LTBI versus TB comparisons, respectively. The AUCs of the other three proteins—BAFF/TNFSF13B, OPN, and LIGHT/TNFSF14 were also significantly higher than a random assignment, 0.700, 0.675, and 0.674, respectively. The highest AUC values for TB vs LTBI differentiation were observed for OPN (AUC = 0.744), BAFF/TNFSF13B (AUC = 0.727), and MMP-2 (AUC = 0.701). In HC vs. TB discrimination the highest AUC values were found in the case of MMP-2 (AUC = 0.848) and BAFF/TNFSF13B (AUC = 0.809), whereas in HC vs. LTBI discrimination the highest AUCs were observed for IL-34 (AUC = 0.722) and MMP-2 (AUC = 0.721) (Table 3).

### 2.4. Identification of the Cytokine Biosignature for Discriminating between Different Status of M.tb Infection

Using the elastic-net logistic regression model, we identified several proteins with sufficient discriminative power between the two studied groups. The serum biosignature consisting of APRIL/TNFSF13, sCD30/TNFRSF8, IFN-α2, IFN-γ, IL-2, sIL-6Rα, IL-8, IL-11, IL-29/IFN-λ1, LIGHT/TNFSF14, MMP-1, MMP-2, MMP-3, osteocalcin, osteopontin, TSLP, and TWEAK/TNFSF12 remained informative of the TB versus LTBI comparison under the 5-fold cross-validation procedure (Table 4). In parallel, in the 5-fold cross-validation analysis between the HC versus LTBI groups, we identified the panel of 15 proteins (IL-6, APRIL/TNFSF13, sCD30/TNFRSF8, gp130/sIL-6β, IL-2, sIL-6Rα, IL-8, IL-29/IFNλ1, IL-35, MMP-2, MMP-3, OPN, PTX-3, sTNF-R2, TWEAK/TNFSF12), while between the HC versus TB groups, 16 proteins (APRIL/TNFSF13, sCD30/TNFRSF8, chitinase 3-like 1, sIL-6Rα, IL-8, IL-11, IL-12(p70), IL-19, IL-28A/IFN-λ2, LIGHT/TNFSF14, MMP-1, MMP-2, MMP-3, OPN, PTX-3, TWEAK/TNFSF12) with the best discriminating potential (Table 4). Additionally, we performed an analysis using an elastic-net multinomial model to compare the three groups in a single model. In consequence, we identified a set of (1) nine predictors informative for the TB group, (2) seven predictors informative for the LTBI group, and (3) thirteen predictors informative for the HC group under the 5-fold cross validation approach. The results are summarized in the Table S3. Interestingly, only sIL-6Rα was informative for all three groups.

### 2.5. Discriminative Biomarker Profiles in Children with a Positive (TST-Positive) or Negative (TST-Negative) Skin Test Reaction to Tuberculin 

**Figure 1:**
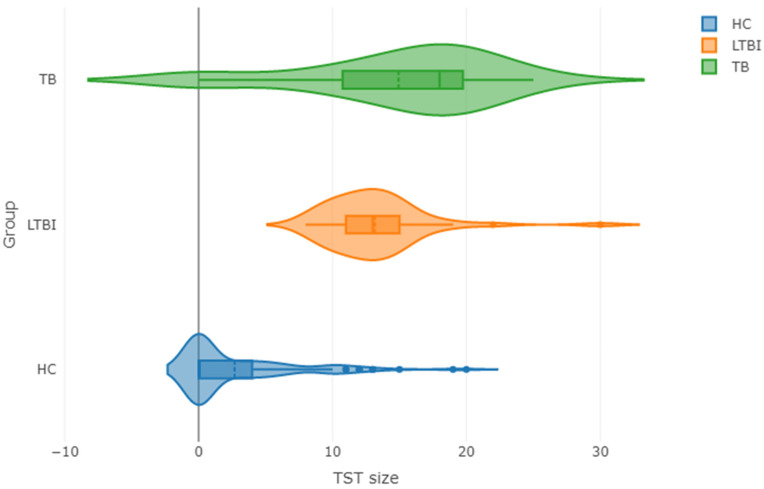
The violin plots for the TST size in the three studied groups. The shape of the plot represents the fitted density of the distribution in the respective group. Abbreviations: HC—*M.tb*-uninfected healthy controls; LTBI—latently *M.tb*-infected individuals; TB—active tuberculosis patients.

We noted that there was a significant difference between the studied groups in the median TST size. At the same time, it must be stressed that there was also a significant difference in the variance of the TST size between the studied groups as tested by the Fligner–Killeen test (*p* = 0.044), which is presented in Figure 1. The analyses of Th-17-related cytokines levels in the sera from TB, LTBI, and HC children with a positive or negative skin reaction to tuberculin showed that the level of TNF-α was significantly higher in the TST-negative individuals compared to the TST-positive children. On the contrary, a significantly lower sCD40L concentration in the sera from the TST-negative in comparison with the TST-positive was noticed in TB patient group (Table S4). The serum levels of the other Th-17-related cytokines between the TST-positive and TST-negative children were comparable. The median concentrations of IFN-α2, IL-12 (p40), IL-22, IL-28A/IFN-λ2, PTX-3, and TSLP were significantly higher in the sera from the TST-negative than TST-positive children from the LTBI group (Table S4). 

We further aimed to find the most informative markers of the TST size regardless of the study group using the elastic-net linear model approach. Interestingly, we found (1) five Th17 associated markers (IL-4, IL-6, IL-31, sCD40L and TNF-α) and (2) twelve inflammation associated proteins (APRIL/TNFSF13, sCD30/TNFRSF8, gp130/sIL-6Rβ, IL-8, IL-10, IL-29/IFN-λ1, IL-35, LIGHT/TNFSF14, MMP-2, Osteopontin, sTNF-R2 and TWEAK/TNFSF12) that are informative of the TST size in the 5-fold cross-validation procedure. The results are summarized in Table 5.

## 3. Discussion

The lack of the gold standard for the diagnosis of *M.tb* infection in children reflects the spurious knowledge on the molecular background of childhood TB, as well as justifies the intensive research on its diagnostic and predictive indicators. The detection of biomarkers in serum or plasma, an alternative to sputum clinical specimens, is thought to be an effective auxiliary method of TB diagnosis in that high-risk group. However, very few studies have been conducted in young children, and increased efforts should be made to develop an accurate and practical biomarker for childhood TB.

In addressing the need for better diagnostic tests allowing the differentiation of different stages of *M.tb* infection in children, the main aim of the study was to select biomarkers of anti-TB protective immunity versus TB disease in children among 53 biological molecules—Th17-related cytokines and inflammatory mediators, whose role in *M.tb* infections has already been suggested. We performed a prospective study in the cohort of 216 BCG-vaccinated Polish children and applied a multiplex quantification approach to compare the serum expression level in uninfected healthy individuals with patients with active TB or *M.tb* latently infected subjects. Using the linear ANOVA approach with 2df, we found that four markers—IL-21, sCD40L, IL-8, and MMP-2 were significantly differentially expressed (under FDR of 0.05) in the comparisons between the HC and jointly analyzed TB and LTBI groups as well as between the TB and LTBI subjects, which confirmed the potential utility of these proteins in distinguishing the state of M.tb infection from the uninfected condition and active from latent TB in children. IL-21 is produced primarily by CD4+ cells and acts on a broad range of immune cells including B and T cells, natural killer cells, dendritic cells, macrophages, and epithelial cells [13]. This cytokine is a critical regulator of immunoglobulin production by B cells and a T cell co-mitogen involved in the expansion of CD8+ T cells. On the other hand, IL-21 also has inhibitory effects on immune responses resulting from the induction of IL-10 by T and B cells and B cell apoptosis. IL-21 together with CD40L induces human B cells to produce IL-10 in memory B cells that have undergone immunoglobulin class switching [14]. IL-10 producing regulatory B10 cells express granzyme B, which degrades the T-cell receptor ζ-chain and limits T-cell proliferation and thus may have a potential for the suppression of immune responses [15]. The elevated serum sCD40L observed in children from the TB group may be also considered to contribute to immunosuppression of adaptive responses. In HIV infection, elevated sCD40L induced immunosuppression by Th17 regulatory T cell expansion [16]. In patients with pancreatic ductal adenocarcinoma, over-expression of circulating sCD40L was found to be correlated with enhanced immunosuppressive cytokine production and a high serum level of IL-8 [17]. In TB children, coexistence of increased sCD40L levels and overproduction of IL-8 was observed. Increased levels of IL-8 and other biomarkers associated with systemic inflammation were demonstrated in plasma from pleural TB patients, whereas pulmonary TB patients without effusions had higher levels of proteins involved in cell mediated immunity, namely, sCD40L and IL-12p40 [18]. Interestingly, our results argue in favor of a role of sCD40L in the occurrence of DTH to tuberculin. A significantly higher sCD40L concentration in the sera from the TST-positive in comparison with the TST-negative TB children was noticed (Table S4). 

Protective immunity to *M.tb* depends firmly on Th17-associated cytokines, mediating both antibacterial and pro-inflammatory host defense mechanisms. They are involved in the processes of activation and recruitment of neutrophils, macrophages, and Th1 lymphocytes into the site of infection, contributing to the delimitation of the damaged area in the lung tissue and the inhibition of *M.tb* growth [19,20]. We found that the children from the TB and LTBI groups manifested reduced serum levels of IL-17A compared to the HC children. It is possible that the genetic variant of the down-regulated IL-17A expression represents an above-average susceptibility to *M.tb* infection. This cytokine plays a role in immunity to intracellular bacteria [21] and is critical for the enhancement of memory responses against these pathogen [22]. A vaccine-induced protection with the BCG vaccine requires the local recruitment of IL-17A-producing T cells [23]. In line with our observations are the results of the meta-analysis by Li et al. on the production of IL-17 in infants or children vaccinated with BCG. The authors indicated that IL-17 levels produced by CD4+ in response to *M.tb* antigens stimulation were lower in pulmonary TB cases compared to healthy controls and healthy tuberculin reactors [24]. Moreover, Kumar et al. showed that pediatric TB was characterized by diminished type Th1, Th2, and Th17 cytokine responses, suggesting a crucial role for these cytokines in the protection against *M.tb* infection [25]. Interestingly, a reduced level of blood IL-17 producing cells was detected also in adult TB [26,27,28]. At the same time, Heidarnezhad et al. found a lower expression of IL-17 and IL-23 mRNA and a lower level of IL-17 producing CD4+ T cells in adults with active TB [28]. On the contrary, some studies reported similar or even elevated plasma levels of Th17-related cytokines in childhood TB [29,30]. Moreover, elevated plasma IL-17 levels, decreasing significantly after TB treatment and smear conversion, were found also in adult pulmonary TB patients [31,32]. Such discrepancies in reported findings may result from heterogeneity of study groups inclusion criteria, age of the patients studied, genetic differences in patient populations, as well as differences in TB disease severity. 

Host inflammatory proteins involved in the regulation of diverse immune mechanisms have been shown to possess a variable potential in the diagnosis of TB in children and adults [11,33,34,35]. One of the most extensively studied non-cytokine markers is matrix metalloproteinases (MMPs), zinc-containing proteases capable of degrading components of pulmonary extracellular matrix [36]. There is growing evidence that the balance existing between different MMPs with similar substrate affinity as well as between MMPs and their inhibitors (TIMPs) may be an important immunoregulatory mechanism in the pathogenesis of TB [37]. Levels of different MMPs have been shown to alternate in the peripheral blood and at the site of infection in both adult and pediatric TB, but their diagnostic significance is still undefined [29,38,39,40]. In our study, serum levels of MMP-2, a 72 kDa type IV collagenase, were significantly lower in the TB children compared to the LTBI or HC individuals. Among the studied proteins, MMP-2 had the highest discriminative potential shown on the basis of the area under the ROC curve (AUC) in both 3-group and 2-group comparisons as well as the cross-validation based elastic-net feature selection. There are several reports on the role of MMP-2 in TB, but none indicate a diagnostic potential of the enzyme in pediatric TB [37,41,42,43,44]. In a cellular model of TB meningitis, Green et al. demonstrated that conditioned medium from *M.tb*-infected primary human monocytes down-regulated the microglial constitutive MMP-2 gene expression and secretion [37]. On the other hand, the expression of MMP-2, as well as MMP-1, -3, -9, -12, -13, and -14 was found to be up-regulated in the lungs of TB-infected rabbits with destructive pathology, and a recent microarray study carried out on a macaque model showed significantly elevated levels of MMP-1, -2, -7, -9, and -14 four weeks after *M.tb* infection [41,42]. Several studies have identified MMPs as markers of acute inflammation in TB [29,45,46,47]. Kumar et al. proposed MMP-1, -7, and -8 and TIMP-1 and -3 as candidates for non-sputum-based biomarkers for active *M.tb* infection in children [29]. MMP-1 and its activator MMP-3 were found to be related to the TB disease severity and treatment efficacy, while MMP-13 together with TIMP-2 were proved to be potential indicators of extra-pulmonary TB in adults [44,45].

Given the moderate size of the study groups, we aimed to perform a more speculative analysis in which we train an elastic-net model to detect the most robust predictors of the study groups. In this analysis, we detected comprehensive panels of several proteins that were specific in discriminating between every two studied groups. The serum biosignature consisting of 16 proteins (APRIL/TNFSF13, sCD30/TNFRSF8, chitinase 3-like 1, sIL-6Rα, IL-8, IL-11, IL-12(p70), IL-19, IL-28A/IFN-λ2, LIGHT/TNFSF14, MMP-1, MMP-2, MMP-3, OPN, PTX-3, TWEAK/TNFSF12) was found informative for the differentiation between the TB and HC groups, while the panel of 15 proteins (IL-6, APRIL/TNFSF13, sCD30/TNFRSF8, gp130/sIL-6β, IL-2, sIL-6Rα, IL-8, IL-29/IFNλ1, IL-35, MMP-2, MMP-3, OPN, PTX-3, sTNF-R2, TWEAK/TNFSF12) distinguished the HC group from the LTBI cohort. Currently, a number of studies have demonstrated a potential of blood-derived host protein biomarkers in the diagnosis of active TB [48,49,50,51,52,53,54,55,56,57,58]. A 6-marker serum protein biosignature consisting of CRP (c-reactive protein), IFN-γ, IP-10 (human interferon-inducible protein 10), CFH (complement factor H), Apo-AI (apolipoprotein AI), and SAA (serum amyloid A) showed a potential in the diagnosis of childhood tuberculous meningitis48. In an adult study, Chen et al. proposed five serum proteins—I-309 (CCL1; chemokine (C-C motif) ligand 1), MIG (CXCL9; chemokine (C-X-C motif) ligand 9), eotaxin-2, IL-8, and ICAM-1 (intercellular adhesion molecule 1) as biomarkers for active TB screening [49]. 

Considering that the currently used immune-based TST and IGRA tests cannot distinguish between latent and active *M.tb* infection, blood-based biomarkers that have this ability would be a real advance for diagnostics. However, the immature immune system of young children, along with dynamic and multi-faceted host-pathogen interactions, makes it difficult to distinguish latent *M.tb* infection from active TB in this age group. In our study, a panel of 17 proteins (APRIL/TNFSF13, sCD30/TNFRSF8, IFN-α2, IFN-γ, IL-2, sIL-6Rα, IL-8, IL-11, IL-29/IFN-λ1, LIGHT/TNFSF14, MMP-1, MMP-2, MMP-3, osteocalcin, osteopontin, TSLP, and TWEAK/TNFSF12) was selected by means of cross-validation to be sufficiently informative of the comparison between the TB versus LTBI children. It follows from the literature data that different models based on the combination of immune mediators, measured either in serum or *M.tb*-stimulated cultures, achieved diagnostic performance to discriminate between active and latent pulmonary TB in children. Chegou et al. reported that the measurement of the levels of IFN-α2, IL-1Ra, sCD40L, and VEGF (vascular endothelial growth factor) might be a useful method for differentiating between active TB disease and latent *M.tb* infection, while other authors found the same utility for IFN-γ, IP-10, ferritin, and 25-hydroxyvitamin D [50,51]. Another six-cytokine signature for detecting TB infection and discriminating active from latent TB included *M.tb* antigen-stimulated levels of IFN-γ, IP-10, and IL-Ra, and unstimulated levels of IP-10, VEGF, and IL-12(p70) [52]. The available data on the potential utility of immune markers in discriminating between active TB and latent infection are conflicting, and the studies conducted to date have used highly heterologous methodology. It should be also assumed that the great variability of cytokine measurements across studies reflecting changes in the balance of plasma cytokine levels is related not only to the progression of *M.tb* infection but also to the levels of other cytokines in the circulation [53].

Assessment of delayed-type hypersensitivity reaction to intradermally administered mycobacterial tuberculin in the tuberculin skin test is used worldwide as a method for diagnosing *M.tb* infection [59]. TST is currently the only assay that allows in vivo testing of responses to mycobacterial antigens and is still considered a useful tool in TB diagnosis; however, high rates of false-positive reactions resulting from the antigenic similarity between BCG, *M.tb*, and environmental nontuberculous mycobacteria lower its diagnostic utility. With this in mind, in our study, we compared the levels of Th-17-related cytokines and inflammatory mediators in the sera from TB, LTBI, and HC children with a positive or negative skin reaction to tuberculin. Our data showed that in all studied groups the level of TNF-α was significantly higher in the TST-negative individuals compared to the TST-positive children. On the contrary, a significantly lower sCD40L concentration in the sera from all the TST-negative groups in comparison with the TST-positive ones was noticed. We further aimed to find the most informative markers of the TST size regardless of the study group using the elastic-net linear model approach. Interestingly, we found five Th17-related markers (IL-4, IL-6, IL-31, sCD40L, and TNF-α) and twelve inflammation associated proteins (APRIL/TNFSF13, sCD30/TNFRSF8, gp130/sIL-6Rβ, IL-8, IL-10, IL-29/IFN-λ1, IL-35, LIGHT/TNFSF14, MMP-2, Osteopontin, sTNF-R2, and TWEAK/TNFSF12) that were informative of the TST size in the 5-fold cross-validation procedure. Our results are consistent with many findings suggesting that the development of tuberculin-driven delayed-type hypersensitivity being a part of the multifaceted host response to *M.tb* is influenced by numerous mediators with pleiotropic inflammatory effects [59,60,61,62].

The limitation of our study is the low number of children with active TB; however, despite this, we were able to detect highly significant differences between the different diagnostic groups. Importantly, we found that combinations of studied proteins had the potential to discriminate not only between the *M.tb*-infected from the uninfected children but also between active TB and latent *M.tb* infection. While these differences may not be sufficient for clinical purposes, the results may prove that some of the studied immune biomarkers may be useful in diagnostic tests. Another weakness of our study is the lack of children with other lung diseases as most of the markers tested have the potential to be expressed similarly also in infections other than those caused by *M.tb*. Future studies involving more children with pulmonary TB, as well as children with extrapulmonary TB, other non-mycobacterial lung diseases, and immunocompromised children are needed to validate the results obtained and confirm the potential of the proposed biosignatures as diagnostic biomarkers for childhood *M.tb* infections.

In summary, our findings indicate that serum-based host immune markers reflect the biological processes associated with *M.tb*—driven host response and have the potential to support the diagnosis of TB or the discrimination between active TB and latent *M.tb* infection in children. They may also provide a new insight into the mechanisms underlying the outcome of *M.tb* infection in children and can subsequently inspire appropriate experimental models aimed at developing new anti-TB vaccines superior to current BCG vaccination and new anti TB drugs.

## 4. Materials and Methods

### 4.1. Children Characteristics

In total, 216 children of both genders (aged 1–15 years) vaccinated with *M. bovis* BCG Moreau were included in the study. All children were examined and diagnosed by infectious disease consultants, including the provincial consultant for the pediatric pulmonology at the Regional Specialized Hospital of Tuberculosis, Lung Diseases and Rehabilitation in Lodz, Poland. The proposed study was approved by the Research Ethics Committee of the Medical University in Lodz (no. RNN/138/15/KE). The pediatricians implementing the project obtained written informed consent from the children’s parents or guardians. All children underwent medical interview, physical examination, and clinical and radiological evaluation including a chest X-ray, tuberculin skin testing, and IGRA testing. In the case of all children with symptoms of lower respiratory tract infections, the differential diagnosis was performed. For this purpose, gastric aspirates or bronchoaspirates collected from the children were examined using standard microbiological methods including Ziehl–Neelsen staining, culturing on liquid (BACTEC MGIT 960 system) media, and genetic testing with the use of the GeneXpert MTB/RIF molecular system. Apart from gastric aspirates or bronchoaspirates, no other extrapulmonary specimens were collected from the children. Based on the complex analysis of the results of the clinical examination, the children were divided into 3 groups: Group 1, including the children with active TB (M.tb culture positive); Group 2 and Group 3, including the healthy children with no signs or symptoms of pulmonary diseases, IGRA positive (LTBI, Group 2) or IGRA negative (Control, Group 3). 

### 4.2. Blood Specimens 

Samples of peripheral blood in a volume of 5 mL were taken from the children prior to the start of the treatment, to prepare serum and perform a whole-blood interferon-gamma assay (QuantiFERON-TB^®^ Gold Plus (QFT), Qiagen, Germany). The QFT assay was conducted according to the manufacturer’s instructions. Briefly, a total of 4 mL of blood was collected in 4 tubes of 1 mL each (Nil control, TB antigen (ESAT-6, CFP-10, TB 7.7)-specific (TB Ag), Mitogen control) followed by a 24 h incubation (370C) and centrifugation (2500 RCF, 15 min), and the concentrations of IFN-γ (interferon-γ) were measured by ELISA. The optical density (OD) of each sample was measured using a multifunctional counter Victor 2 (Wallac Oy, Turku, Finland) fitted with a 450 nm filter. The data were processed and interpreted using the calculation QuantiFERON-TB Gold Analysis Software supplied with the kit. The test result was considered positive if the IFN-gamma level in the sample tube after stimulation with TB Ag was 0.35 IU/mL (after subtraction of the value for the Nil tube). A positive test result (IGRA(+)) was interpreted as latent M.tb infection, whereas a negative IGRA (IGRA(–)) result meant no infection with M.tb. The examination of all IGRA(–) cases was repeated 6 weeks after the first blood donation.

### 4.3. Tuberculin Skin Testing 

The tuberculin skin test was performed using 2 tuberculin units (TU) of the purified protein derivative (PPD) RT23 (Statens Serum Institute, Copenhagen, Denmark) and the Mantoux technique. The diameter of skin induration was measured after 48–72 h by experienced staff. A result was considered positive when the size of induration was equal to or greater than 10 mm.

### 4.4. Multiplex Bead Assays 

Fifty-two cytokines, chemokines, and inflammation mediators were measured simultaneously in sera samples and processed according to the specifications of a Bio-Plex Pro™ Human Th17 assay [interleukin (IL): IL-1β, IL-4, IL-6, IL-10, IL-17A, IL-17F, IL-21, IL-22, IL-23, IL-25, IL-31, IL-33, IFN-γ, sCD40L (soluble CD40 ligand), TNF-α (tumor necrosis factor-α)], and a Bio-Plex Human Inflammation Panel 1 assay [APRIL/TNFSF13 (proliferation-inducing ligand/TNF ligand superfamily member 13), BAFF/TNFSF13B (B-cell activating factor TNFSF13B), sCD30/TNFRSF8 (sCD30/TNF receptor SF8), sCD163 (soluble macrophage activating factor), chitinase 3-like 1, gp130/sIL-6Rβ (soluble IL-6 signal transducer sIL-6Rβ/gp130), IFN-α2, IFN-β, IFN-γ, IL-2, sIL-6Rα, IL-8, IL-10, IL-11, IL-12 (p40), IL-12 (p70), IL-19, IL-20, IL22, IL-26, IL-27 (p28), IL-28A/IFN-λ2, IL-29/IFN-λ1, IL-32, IL-34, IL-35, LIGHT/TNFSF14, MMP-1 (matrix metalloproteinase-1), MMP-2, MMP-3, osteocalcin, osteopontin (OPN), pentraxin-3 (PTX3), sTNF-R1 (soluble TNF-receptor 1), sTNF-R2, TSLP (thymic stromal lymphopoietin), TWEAK/TNFSF1 (TNF-like weak inducer of apoptosis/TNFSF1)], purchased from Bio-Rad (Hercules, CA, USA). In brief, 50 µL of mixed beads was added to prewet wells and washed twice. After the addition of 50 µL of a standard, in-house control or a sample, the plate was incubated for one hour. After the subsequent washing, 25 µL of a detection antibody mixture was added to each well and the plate was incubated for 30 min and then washed. In the next step, 50 µL of streptavidin-PE was added, and after 10 min incubation and washing of the plate, the beads were finally re-suspended in 125 µL assay buffer. Assay readings were made using a Bio-Plex MAGPIXTM Multiplex Reader (Bio-Rad) with the Bio-Plex manager 5.0 software (Bio-Rad version 5.0). Standard samples were analyzed using a Five-Parameter Logistic (5PL) regression curve fitting (Bio-plex software). For each cytokine the standard curve ran from 3.2 to 10,000 pg/mL.

### 4.5. Statistical Analysis 

Statistical analysis was performed using GraphPad Prism version 8 (GraphPad Software, San Diego, CA, USA; http://www.graphpad.com accessed on 15 November 2020) and R Version 3.6.0 (R, Vienna, Austria; http://www.R-project.org accessed on 15 November 2020). Non-parametric tests were used to compare protein levels between the diagnostic groups: Mann–Whitney U test for two-group comparisons and Kruskal–Wallis tests for multiple groups. Categorical variables were compared using two-tailed chi-square tests. For basic differential expression, the limma package was used. The protein expression levels were quantile normalized prior to testing. In linear models, we considered the expression level to be the dependent variable and the group assignment to be the independent variable. We did not control for additional covariates in order to avoid overfitting. To increase the power of the study, we used orthogonal contrasts between the three groups with: the first contrast measuring the difference between HC and (jointly) TB and LTBI, whereas the second contrasts measuring the difference between TB and LTBI. The ROC analysis was performed with the aid of package pROC in R, with the 3-class AUC estimated via the function “multiclass.roc”. The most informative sets of features (protein expression profiles) were detected in a logistic model of penalized regression (elastic-net) by means of 5-fold cross-validation. We fitted regression models separately for Th17-related cytokines and inflammatory mediators due to limited sample size. We consider only binomial (not multinomial) models as the sample size did not guarantee robust results. Hence, we considered three different (in theory independent) models: HC vs TB, HC vs LTBI and LTBI vs TB. For the TST, only one model for Th17-related cytokines and one for inflammatory mediators was fitted with the same methods as above used for identification of informative features. The elastic-net models were fitted with the aid of ‘glmnet’ package in R. The violin plots were made with the ggplot2 package in R.

## Figures and Tables

**Table 1:** General information for studied groups.

Parameter	Groups		
	TB	LTBI	HC
N	15	50	151
Sex M/F	5/10	24/26	89/62
Ethnicity	Caucasian	Caucasian	Caucasian
Age			
median	15	8	7
range	1–17	1–17	1–17
years (IQR)	11–16	5–12	3–11
BCG vaccination	100%	100%	100%
QFT result, N (%)			
positive	12 (80%)	50 (100%)	0 (0%)
negative	3 (20%)	0 (0%)	151 (100%)
TST result, N (%)			
positive	13 (87%)	44 (88%)	18 (12%)
negative	2 (13%)	6 (12%)	133 (88%)
WBC, Counts/mm^3^	9770	8130	8550
Neutrophils (%)	60.7	43.8	40.1
Lymphocytes (%)	26.8	42.8	47.4
Monocytes (%)	9.2	7.8	8.1
Eosinophils (%)	2.6	4.9	3.8
Basophils (%)	0.5	0.5	0.6
RBC, Counts/mm^3^	4,630,000	4,730,000	4,790,000
HGB, g/dL	13.1	13.3	13.2
HCT, %	38.9	39.5	39.2
MCHC, g/dL	33.6	33.8	33.7
PLT, Counts/mm^3^	348,300	307,800	309,800
Bilirubin (mg/dL)	0.3	0.4	0.3
ALT (U/L)	12.1	19.7	15.6
AST (U/L)	18.9	25.7	27.5
CRP (mg/L)	41.9	0.7	0.8

Abbreviations: ALT—alanine aminotransferase; AST—aspartate aminotransferase; BCG—Bacille Calmette–Guerin; CRP—c-reactive protein; HC—*M.tb*-uninfected healthy controls; HCT—hematocrit; HGB—hemoglobin; LTBI—latently *M.tb*-infected individuals; MCHC—mean corpuscular hemoglobin concentration; PLT—platelets; RBC—red blood cells; QFT—QuantiFERON TB Gold test; TB—active tuberculosis patients; TST—tuberculin skin test; WBC—white blood cells.

**Table 2:** The results of a linear model-based approach where two differences are tested between HC and TB and LTBI (jointly), and between TB and LTBI.

Proteins	Coefficients		*p* Value	Adjusted *p* Value
	HC vs. TB and LTBI (Jointly)	TB vs. LTBI		
Th-17-Related Cytokines				
IL-21	−1.83 × 10^2^	−1.09 × 10^2^	<0.001	<0.001
sCD40L	−3.33 × 10^3^	6.92 × 10^2^	0.003	0.025
IL-10	38.3	−8.04	0.034	0.173
IL-6	−2.67 × 10^2^	−1.07 × 10^2^	0.052	0.196
IL-31	1.09 × 10^3^	5.58 × 10^2^	0.148	0.444
IL-17A	60.6	−4.14	0.177	0.444
IL-23	1.15 × 10^3^	−7.61 × 10^2^	0.253	0.543
TNF-α	3.59 × 10^2^	−42.5	0.349	0.640
IL-33	3.67 × 10^2^	−30.0	0.427	0.640
Inflammatory markers				
MMP-2	4.42 × 10^3^	−1.31 × 10^3^	<0.001	0.003
IL-8	−1.31 × 10^3^	9.64 × 10^2^	0.003	0.057
sCD163	−4.48 × 10^10^	4.48 × 10^10^	0.012	0.151
LIGHT/TNFSF14	−33.7	28.8	0.028	0.261
IL-34	−3.35	−0.513	0.044	0.326
chitinase 3-like 1	−1.37 × 10^3^	3.57 × 10^2^	0.056	0.350
sTNF-R1	−4.84 × 10^2^	1.06 × 10^2^	0.075	0.401
IL-12 (p70)	−1.93	1.87	0.111	0.455
IFN-γ	14.5	−5.05	0.117	0.455
OPN	−94.5	−2.49 × 10^3^	0.123	0.455

Abbreviations: HC—*M.tb*-uninfected healthy controls; LTBI—latently *M.tb*-infected individuals; TB—active tuberculosis patients.

**Table 3:** Results of the ROC curve analyses of studied proteins.

Protein	Comparisons			
	3-Class AUC	2-Class AUC		
		HC vs. TB	HC vs. LTBI	LTBI vs. TB
Th-17-related cytokines				
IL-1β	0.554	0.562	0.570	0.530
IL-4	0.570	0.524	0.603	0.583
IL-6	0.556	0.594	0.530	0.544
IL-10	0.614	0.668	0.479	0.694
IL-17A	0.611	0.647	0.567	0.620
IL-17F	0.517	0.453	0.564	0.534
IL-21	0.580	0.632	0.655	0.453
IL-22	0.502	0.484	0.510	0.512
IL-23	0.608	0.658	0.649	0.518
IL-25	0.530	0.422	0.531	0.638
IL-31	0.536	0.545	0.447	0.618
IL-33	0.524	0.538	0.548	0.486
IFN-γ	0.559	0.584	0.534	0.558
sCD40L	0.615	0.676	0.605	0.565
TNF-α	0.563	0.587	0.505	0.598
Inflammatory mediators				
APRIL/TNFSF13	0.519	0.529	0.516	0.512
BAFF/TNFSF13B	0.700	0.809	0.562	0.727
sCD30/TNFRSF8	0.526	0.609	0.421	0.546
sCD163	0.580	0.555	0.563	0.620
chitinase 3-like 1	0.661	0.722	0.637	0.623
gp130/sIL-6Rβ	0.369	0.298	0.419	0.388
IFN-α2	0.511	0.523	0.451	0.556
IFN-β	0.541	0.536	0.526	0.559
IFN-γ	0.580	0.654	0.621	0.463
IL-2	0.518	0.522	0.536	0.494
sIL-6Rα	0.454	0.429	0.506	0.424
IL-8	0.661	0.761	0.521	0.700
IL-10	0.524	0.471	0.540	0.559
IL-11	0.383	0.320	0.442	0.387
IL-12 (p40)	0.506	0.505	0.529	0.483
IL-12 (p70)	0.567	0.530	0.577	0.591
IL-19	0.473	0.458	0.482	0.479
IL-20	0.563	0.603	0.509	0.576
IL-22	0.522	0.530	0.531	0.504
IL-26	0.505	0.507	0.489	0.519
IL-27	0.558	0.578	0.513	0.582
IL-28A/IFN-λ2	0.530	0.546	0.497	0.544
IL-29/IFN-λ1	0.529	0.470	0.590	0.526
IL-32	0.581	0.552	0.577	0.613
IL-34	0.636	0.580	0.722	0.603
IL-35	0.570	0.570	0.600	0.539
LIGHT/TNFSF14	0.674	0.736	0.661	0.626
MMP-1	0.527	0.541	0.516	0.522
MMP-2	0.758	0.848	0.721	0.701
MMP-3	0.532	0.558	0.466	0.570
Osteocalcin	0.404	0.355	0.444	0.412
OPN	0.675	0.738	0.543	0.744
PTX-3	0.539	0.664	0.565	0.387
sTNF-R1	0.588	0.619	0.620	0.524
sTNF-R2	0.606	0.692	0.480	0.645
TSLP	0.484	0.472	0.483	0.495
TWEAK/TNFSF12	0.536	0.685	0.544	0.378

Abbreviations: HC—*M.tb*-uninfected healthy controls; LTBI—latently *M.tb*-infected individuals; TB—active tuberculosis patients.

**Table 4:** The results of 3 elastic-net logistic regression models. The coefficients represent relative differences in serum expression of respective proteins between the studied groups (under the optimal lambda parameter for the penalty function). The most informative set of markers was chosen based on the 5-fold cross-validation approach. The dashes correspond to non-informative predictors.

Proteins	HC vs. LTBI	HC vs. TB	TB vs. LTBI
Th17-related cytokines			
IL-1β	-	-	-
IL-4	-	-	-
IL-6	3.87 × 10^−4^	-	-
IL-10	-	-	-
IL-17A	-	-	-
IL-17F	-	-	-
IL-21	-	-	-
IL-22	-	-	-
IL-23	-	-	-
IL-25	-	-	-
IL-31	-	-	-
IL-33	-	-	-
IFN-γ	-	-	-
sCD40L	-	-	-
TNF-α	-	-	-
Inflammatory mediators			
April/TNFSF13	9.43 × 10^−7^	5.90 × 10^−6^	1.81 × 10^−6^
BAFF/TNFSF13B	-	-	-
sCD30/TNFRSF8	−7.60 × 10^−4^	−1.36 × 10^−3^	−1.07 × 10^−3^
sCD163	-	-	-
chitinase 3-like 1	-	5.41 × 10^−4^	-
Gp130/sIL-6Rbeta	3.67 × 10^−5^	-	-
IFN-α2	-	-	−1.45 × 10^−1^
IFN-γ	-	-	−9.30 × 10^−3^
IL-2	−3.24 × 10^−4^	-	1.52 × 10^−4^
sIL-6Rα	4.41 × 10^−5^	−4.18 × 10^−4^	−3.25 × 10^−4^
IL-8	2.94 × 10^−4^	1.60 × 10^−4^	2.41 × 10^−4^
IL-10	-	-	-
IL-11	-	−1.13 × 10^−2^	−3.68 × 10^−3^
IL-12 (p40)	-	-	-
IL-12 (p70)	-	−4.71	-
IL-19	-	−1.05 × 10^−1^	-
IL-27	-	-	-
IL-28A/IFNλ2	-	−1.45 × 10^−4^	-
IL-29/IFNλ1	2.18 × 10^−2^	-	−3.50 × 10^−2^
IL-32	-	-	-
IL-34	-	-	-
IL-35	4.26 × 10^−3^	-	-
LIGHT/TNFSF14	-	6.50 × 10^−2^	7.13 × 10^−3^
MMP-1	-	7.36 × 10^−5^	6.44 × 10^−5^
MMP-2	−1.51 × 10^−4^	−4.09 × 10^−4^	−3.55 × 10^−4^
MMP-3	−8.31 × 10^−5^	3.72 × 10^−3^	4.46 × 10^−3^
osteocalcin	-	-	6.23 × 10^−7^
OPN	6.75 × 10^−5^	−5.10 × 10^−4^	−5.81 × 10^−5^
PTX-3	−2.44 × 10^−4^	−1.44 × 10^−4^	-
sTNFR1	-	-	-
sTNFR2	−1.70 × 10^−4^	-	-
TSLP	-	-	−1.28 × 10^−2^
TWEAK/TNFSF12	−1.93 × 10^−3^	−1.37 × 10^−2^	−6.15 × 10^−3^

Abbreviations: HC—*M.tb*-uninfected healthy controls; LTBI—latently *M.tb*-infected individuals; TB—active tuberculosis patients.

**Table 5:** The results of the elastic-net linear regression model. The coefficients represent association between the serum expression of respective proteins and TST (under the optimal lambda parameter for the penalty function). The most informative set of markers was chosen based on the 5-fold cross-validation approach. The blank spaces correspond to non-informative predictors.

Th17-Related Cytokines	Coefficient	Inflammatory Mediators	Coefficient
IL-1β	-	April/TNFSF13	1.37 × 10^−7^
IL-4	−3.21 × 10^−5^	BAFF/TNFSF13B	-
IL-6	2.68 × 10^−4^	sCD30/TNFRSF8	−6.09 × 10^−4^
IL-10	-	sCD163	-
IL-17A	-	chitinase 3-like 1	-
IL-17F	-	gp130/sIL-6Rβ	−3.57 × 10^−6^
IL-21	-	IFN-α2	-
IL-22	-	IFN-γ	-
IL-23	-	IL-2	-
IL-25	-	sIL-6Rα	-
IL-31	−1.57 × 10^−5^	IL-8	3.22 × 10^−5^
IL-33	-	IL-10	1.02 × 10^−3^
IFN-γ	-	IL-11	-
sCD40L	3.11 × 10^−6^	IL-12 (p40)	-
TNF-α	−9.67 × 10^−5^	IL-12 (p70)	-
		IL-19	-
		IL-27	-
		IL-28A/IFNλ2	-
		IL-29/IFNλ1	6.86 × 10^−4^
		IL-32	-
		IL-34	-
		IL-35	1.68 × 10^−3^
		LIGHT/TNFSF14	1.29 × 10^−4^
		MMP-1	-
		MMP-2	−7.76 × 10^−5^
		MMP-3	-
		osteocalcin	-
		OPN	7.65 × 10^−6^
		PTX-3	-
		sTNFR1	-
		sTNFR2	−1.77 × 10^−4^
		TSLP	-
		TWEAK/TNFSF12	−6.58 × 10^−4^

Abbreviations: HC—*M.tb*-uninfected healthy controls; LTBI—latently *M.tb*-infected individuals; TB—active tuberculosis patients.

## Data Availability

The data presented in this study are available on request from the corresponding author. The data are not publicly available due to ethical restrictions.

## Supplementary Materials

The following are available online at https://www.mdpi.com/article/10.3390/pathogens10050517/s1, Table S1: Median concentrations of Th17-related cytokines and inflammatory mediators in children’s sera. Table S2: The results of a linear model-based approach where three differences are tested: (1) between the HC and LTBI, (2) between HC and TB, and (3) between TB and LTBI. Table S3: The results of elastic-net multinomial regression model. The coefficients represent relative differences of serum expression of respective proteins between studied groups (under the optimal lambda parameter for the penalty function). The most informative set of markers was chosen based on the 5-fold cross-validation approach. The dashes correspond to non-informative predictors. Table S4: Levels of Th-17-related cytokines and inflammatory mediators in the sera from TB, LTBI, and HC children with positive (TST-positive) or negative (TST-negative) skin reaction to tuberculin.
