# Supplementary material for: Cytokine Biosignature of Active and Latent Mycobacterium Tuberculosis Infection in Children
Source: Pathogens. 2021 Apr 24;10(5):517. doi: 10.3390/pathogens10050517 (PMC8145955; doi:10.3390/pathogens10050517)
Supplement: Supplementary file 1 [file pathogens-10-00517-s001.zip › pathogens-1190168-supplementary.pdf]

Supplementary information

Cytokine biosignature of active and latent *Mycobacterium tuberculosis* infection in children

Magdalena Druszczynska, Michal Seweryn, Sebastian Wawrocki, Magdalena Kowalewska-Pietrzak, Anna Pankowska, Wieslawa Rudnicka

Supplementary Table S1. Median concentrations of Th17-related cytokines and inflammatory mediators in children's sera

Supplementary Table S2. The results of a linear model-based approach where three differences are tested: (1) between the HC and LTBI, (2) between HC and TB, and (3) between TB and LTBI.

Supplementary Table S3. The results of elastic-net multinomial regression model. The coefficients represent relative differences of serum expression of respective proteins between studied groups (under the optimal lambda parameter for the penalty function). The most informative set of markers was chosen based on the 5-fold cross-validation approach. The dashes correspond to non-informative predictors.

Supplementary Table S4. Levels of Th-17-related cytokines and inflammatory mediators in the sera from TB, LTBI and HC children with positive (TST-positive) or negative (TST-negative) skin reaction to tuberculin.

Supplementary Table S1. Median concentrations of Th17-related cytokines and inflammatory mediators in children’s sera

Protein	TB	LTBI	HC
	Me; IQR (pg/ml)	Me; IQR (pg/ml)	Me; IQR (pg/ml)
Th-17-related cytokines			
IL-4	0; 0-57.7	0; 0-27.4	0; 0-50.6
IL-6	15.1; 3.8-35.7	2.14; 0-12.7	5.29; 0-24.8
IL-10	0; 0-25.0	0; 0-17.0	6.88; 0-34.5
IL-17F	17.5; 0-90.5	0; 0-41.0	5.34; 0-59.6
IL-21	46.4; 0-95.9	38.9; 0-56.2	4.26; 0-67.5
IL-22	7.22; 0-78.0	0; 0-39.6	0; 0-68.8
IL-25	0; 0-20.1	0; 0-11.1	4.8; 0-26.6
IL-31	725.0; 540.0-1133.0	680.0; 379.0-1226.0	822.0; 389.0-1228.0
IL-33	32.5; 12.9-215.0	30.2; 3.56-156.0	31.1; 0-272.0
IFN-γ	2.65; 0-42.2	4.88; 0-53.5	5.43; 0.66-121.0
sCD40L	2979.0; 1251.0-3562.0	2565.0; 1076.0-4688.0	1139.0; 538.0-3584.0
TNF-α	36.4; 19.9-108.0	30.3; 16.1-101.0	40.3; 18.6-101.0
IL-1β	4.97; 0-39.7	0[a]; 0-16.7	6.68[a]; 0-44.0
IL-17A	0; 0-7.92	0[b]; 0-15.9	4.01[b]; 0-24.3
IL-23	24.5; 0-125.0	10.4[c]; 0-178.0	67.2[c]; 6.81-205.0
Inflammatory mediators			
APRIL/TNFSF13	864686[d/e]; 493031-1006100	439681[d;] 301873-816166	379919[e]; 287429-525611
BAFF/TNFSF13B	2412; 330-4834	1201; 631-3593	1195; 929-1634
sCD30/TNFRSF8	201; 53.1-513	320; 174-463	372; 270-555
sCD163	0.43; 0.19-2.88	0.19; 0.06-2.52	0.29; 0.07-0.98
Chitinase 3-like 1	3020; 1145-3723	2991; 1722-3666	2343; 1478-3494
gp130/sIL-6R	31926; 25440-33901	33448; 28523-40262	32038; 30185-35883
IFN-α2	3.03; 1.39-3.92	3.14; 2.52-4.67	3.03; 2.52-3.77
IFN-β	2237; 1898-2372	2245; 2130-2336	2211; 2141-2294
IL-2	2682; 2379-2718	2659; 2609-2743	2665; 2567-2735
sIL-6Rα	1376; 677-2428	1888; 1215-3703	2223; 1305-2967
IL-8	30.6; 6.92-398	8.29; 4.47-46.6	9.04; 5.13-43.4
IL-10	0.45; 0.01-0.6	0.54; 0.34-0.84	0.50; 0.27-0.70
IL-11	173; 80.6-192	165; 151-196	167; 144-188
IL-12 (p40)	7.82; 3.5-9.95	8.99; 7.21-11.0	8.25; 6.91-10.7
IL-12 (p70)	0.03; 0.01-0.04	0.04; 0.02-0.07	0.03; 0.02-0.04
IL-19	6.4; 3.4-9.7	7.69; 6.4-9.1	7.1; 5.9-8.2
IL-20	>5810	>5810	>5810
IL-22	5.98; 2.74-8.62	6.29; 4.24-9.33	5.67; 4.56-7.30
IL-26	3.72; 2.16-4.29	4.21; 3.49-4.97	4.13; 3.51-4.46
IL-27	5.64; 0-14.2	9.83; 3.63-20.8	7.71; 2.9-13.6
IL-28A/IFN-λ2	0; 0-1813	1065; 0-2508	1065; 0-2070
IL-29/IFN-λ1	14; 6.33-26.7	19.5; 12.3-25.6	14.4; 11.5-21.7
IL-32	0	0	0
IL-34	0; 0-1	0.45; 0-1.98	0; 0-0.18
IL-35	0; 0-5.95	0; 0-5.95	0; 0-1.99

LIGHT/TNFSF14	3.62; 0-19.2	1.17; 0-4.24	0.15; 0-2.61
MMP-1	28498; 13959-38039	25248; 20055-32649	24872; 20342-28479
MMP-2	1061[f/g]; 350-3589	3230[f]; 1651-5057	4541[g]; 3334-6087
MMP-3	369; 161-720	286; 187-418	310; 206-494
osteocalcin	>208184	>208184	>208184
osteopontin (OPN)	1867[h/i]; 634-3287	4370[h]; 1936-5738	3623[i]; 2377-5125
pentraxin-3 (PTX-3)	5235[j]; 4452-6435	6033; 5291-6639	6329[j]; 5531-7155
sTNF-R1	400; 111-970	546; 277-802	573; 373-763
sTNF-R2	254; 0-622	326; 149-567	312; 157-736
TSLP	25.4; 6.13-34.6	26.8; 19.8-42.9	24.6; 17.8-33.8
TWEAK/TNFSF12	56.7; 13.8-102	95.4; 61.1-140	78.2; 49.2-116

Abbreviations: TB – tuberculosis children; LTBI – children with latent *M.tb* infection, HC – healthy controls; Me – median, IQR – interquartile range.
[a]p=0.035, [b]p=0.023, [c]p=0.031,[d]p=0.03; [e]p=0.0005; [f]p=0.02; [g]p=0.0001; [h]p=0.004; [i]p=0.02; [j]p=0.03

Supplementary Table S2. The results of a linear model-based approach where three differences are tested: (1) between the HC and LTBI, (2) between HC and TB, and (3) between TB and LTBI.

Proteins	Coefficients			p value	adjective p value
	HC versus LTBI	HC versus TB	TB versus LTBI		
Th-17-related cytokines					
IL-21	-1.46E+02	-3.70E+01	-1.09E+02	p<0.001	p<0.001
sCD40L	-1.32E+03	-2.01E+03	6.92E+02	0.003	0.025
IL-10	1.51E+01	2.32E+01	-8.04E+00	0.034	0.174
IL-6	-1.87E+02	-8.01E+01	-1.07E+02	0.052	0.196
IL-31	8.22E+02	2.64E+02	5.58E+02	0.148	0.445
IL-17A	2.82E+01	3.24E+01	-4.14E+00	0.178	0.445
IL-23	1.96E+02	9.57E+02	-7.61E+02	0.254	0.543
TNF-α	1.58E+02	2.01E+02	-4.25E+01	0.349	0.641
IL-33	1.69E+02	1.99E+02	-3.00E+01	0.427	0.641
Inflammatory mediators					
MMP-2	1.55E+03	2.87E+03	-1.31E+03	p<0,001	0.003
IL-8	-1.71E+02	-1.13E+03	9.64E+02	0.003	0.057
sCD163	-1.04E+03	-4.48E+10	4.48E+10	0.012	0.151
LIGHT/TNFSF14	-2.46E+00	-3.13E+01	2.88E+01	0.028	0.261
IL-34	-1.93E+00	-1.42E+00	-5.13E-01	0.044	0.326
chitinase 3-like 1	-5.05E+02	-8.61E+02	3.57E+02	0.056	0.350
sTNF-R1	-1.89E+02	-2.95E+02	1.06E+02	0.075	0.401
IL-12 (p70)	-2.79E-02	-1.90E+00	1.87E+00	0.111	0.455
IFN-γ	4.72E+00	9.77E+00	-5.05E+00	0.117	0.455
osteopontin	-1.29E+03	1.20E+03	-2.49E+03	0.123	0.455

Supplementary Table S3. The results of elastic-net multinomial regression model. The coefficients represent relative differences of serum expression of respective proteins between studied groups (under the optimal lambda parameter for the penalty function). The most informative set of markers was chosen based on the 5-fold cross-validation approach. The dashes correspond to non-informative predictors.

	TB	LTBI	HC
Th17-related cytokines			
IL-1β	-	-	-
IL-4	-	-	-
IL-6	-	-	-
IL-10	-	-	-
IL-17A	-	-	-
IL-17F	-	-	-
IL-21	-	-	-
IL-22	-	-	-
IL-23	-	-	-
IL-25	-	-	-
IL-31	-	-	-
IL-33	-	-	-
IFN-γ	-	-	-
sCD40L	-	-	-
TNF-α	-	-	-
Inflammatory mediators-			
April/TNFSF13	1.80E-02	-	-1.32E-03
BAFF/TNFSF13B	-	-	-
sCD30/TNFRSF8	-8.51E-03	-	9.92E-03
sCD163	-	-	-
Gp130/sIL-6Rbeta	-	-	-5.72E-03
IFN-alfa2	-	3.13E-01	-3.82E-03
IFN-gamma	-	-	-
IL-2	-	-	-
sIL-6Ralfa	-2.58E-03	4.26E-02	3.03E-03
IL-8	2.23E-04	-	3.65E-02
IL-10	-	-	-
IL-11	-	-	-7.29E-05
IL-12 (p40)	-	-	-
IL-12 (p70)	-	-	-
IL-19	-	-	-
IL-27	-	-	-
IL-28A/IFN lambda2	-	-	2.65E-01
IL-29/IFNlambda1	-	4.27E-02	-
IL-34IL-35	-	-	-
IL-35	-	-	-
LIGHT/TNFSF14	-	-	-
MMP-1	4.96E-02	-	-2.68E-02
MMP-2	-2.79E-02	-	2.26E-02
MMP-3	2.76E-02	-2.10E-02	-
osteopontin (OPN)	-2.35E-03	1.13E-02	-
Pentraxin 3	-	-	2.94E-02
sTNFR1	-	-	-
sTNFR2	-	-4.60E-03	1.20E-03
TSLP	-	1.49E-03	-
TWEAK/TNFSF12	-3.55E-03	-	4.76E-03

Supplementary Table S4. Levels of Th-17-related cytokines and inflammatory mediators in the sera from TB, LTBI and HC children with positive (TST-positive) or negative (TST-negative) skin reaction to tuberculin.

Protein	Concentration<br>Me (IQR)					
	TB		LTBI		HC	
	TST-positive	TST-negative	TST-positive	TST-negative	TST-positive	TST-negative
Th-17-related cytokines						
IL-4	0<br>0-59.30	0<br>0	0<br>0-31.10	0<br>0-28.7	0<br>0-48.20	0<br>0-51
IL-6	14.5<br>3.79-30.6	43.8<br>16.9-70.6	0.965<br>0-12.4	6.51<br>0.72-40.20	2.86<br>0-22.5	5.84<br>0.24-29.40
IL-10	0<br>0-25.5	8.88<br>8.23-9.53	0<br>0-21.0	1.12<br>0-9.94	9.75<br>0-44.10	6.17<br>0-32.40
IL-17F	1.05<br>0-92.9	33.5<br>17.5-49.5	0<br>0-54.2	18.1<br>0-30.5	2.67<br>0-72.8	5.34<br>0-59.5
IL-21	46.4<br>0-93.7	75.7<br>4.26-147.0	40.4<br>0-57	16.9<br>4.1-56.6	29.8<br>0-94.7	4.26<br>0-65.6
IL-22	0<br>0-88.9	14.9<br>7.22-22.6	0<br>0-42.7	0<br>0-29.2	0<br>0-51.8	1.11<br>0-74.2
IL-25	0<br>0-17.8	17.8<br>6.45-29.1	0<br>0-13.9	0.795<br>0-5.27	4.79<br>0-29.2	5.36<br>0-24.6
IL-31	679<br>519-914	1967<br>1256-2677	680<br>136-1279	645<br>491-1037	861<br>34.6-976	804<br>399-1304
IL-33	32.5<br>13.9-217	82<br>0-164	25.3<br>3.56-291	46<br>13.7-115	78.4<br>0-368	31.1<br>0-268
IFN-γ	3.72<br>0-81.7	1.84<br>1.02-2.65	1.91<br>0-50	31.3<br>3.02-76.7	16.9<br>1.04-160	2.49<br>0.64-120
sCD40L	3070[a]<br>1940-4368	794[a]<br>720-868	2473<br>916-4712	3620<br>1592-4192	963<br>404-3782	1139<br>538-3446
TNF-α	35.4[b]<br>15.6-93.9	73.3[b]<br>38.4-108	30.3[c]<br>13.5-82.1	61.9[c]<br>23.2-133	20.2[d]<br>0.47-69	42.1[d]<br>21.9-156
IL-1β	3.54<br>0-31.4	91.3<br>33.5-149.0	0<br>0-12.8	20.3<br>1.47-96.3	1.06<br>0-22.6	5.76<br>0-47.4
IL-17A	0<br>0-6.53	27.5<br>7.92-47.2	0<br>0-22.2	0<br>0-3.97	2.69<br>0-18.3	4.3<br>0-24.3
IL-23	15.5<br>0-111	226<br>119-333	10.4<br>0-243	7.73<br>0-47.3	41.2<br>5.66-194	67.2<br>6.83-213
Inflammatory mediators						
APRIL/TNFSF<br>13	842327<br>470010-956723	1147159<br>944618-1349700	479407<br>372325-816472	331262<br>120142-737397	286638<br>223971-422787	386665<br>297992-567490
BAFF/TNFSF1<br>3B	1506<br>327-3938	7689<br>3989-11389	1175<br>617-2127	4601<br>852-23118	1532<br>737-1626	1188<br>933-1771
sCD30/TNFRS<br>F8	177<br>46.6-334	533<br>513-553	320<br>170-458	380<br>231-629	413<br>381-557	346<br>252-558
Chitinase 3-like<br>1	3020<br>754-3565	4346<br>1319-7373	2475<br>1694-3712	3080<br>2172-4559	3132<br>1650-4391	2042<br>1424-3493
gp130/sIL-6R	31631<br>22954-33298	110144<br>32503-187786	33329<br>28420-40658	33448<br>29452-37300	34131<br>33967-36255	31603<br>30115-35557
IFN-α2	3.03<br>1.30-3.74	3.33<br>2.74-3.92	3.08[e]<br>2.45-3.95	5.45[e]<br>3.48-6.41	3.32<br>2.97-3.43	2.97<br>2.49-4.05
IFN-β	2237<br>1879-2346	2277<br>2179-2376	2219<br>2125-2317	2409<br>2240-2429	2245<br>2055-2283	2201<br>2141-2302
IL-2	2682<br>2363-2714	2672<br>2609-2735	2645<br>2605-2732	2786<br>2652-2825	2698<br>2540-2707	2665<br>2571-2742
sIL-6Rα	1376<br>403-2282	2576<br>1359-3793	1855<br>1180-3719	2193<br>1717-3179	2983<br>1998-3701	2015<br>1279-2916
IL-8	13.10<br>6.92-254	571.00<br>289-853	9.82<br>4.31-38.4	8.29<br>4.81-3131	5.13<br>3.6-18.8	10.10<br>6.14-45.8
IL-10	0.45<br>0.005-0.64	0.47<br>0.34-0.6	0.52<br>0.24-0.77	1.1<br>0.48-5.81	0.49<br>0.14-0.6	0.52<br>0.3-0.74
IL-11	173<br>40.3-196	166<br>154-179	163<br>148-196	179<br>163-208	167<br>147-180	168<br>143-190
IL-12 (p40)	7.82<br>3.24-10.1	8.30<br>7.37-9.23	8.45[f]<br>6.84-10.2	12.30[f]<br>10.3-25.6	8.45<br>6.61-8.6	8.21<br>6.91-11
IL-12 (p70)	0.03<br>0.01-0.04	0.06<br>0.02-0.11	0.03<br>0.02-0.07	0.06<br>0.04-0.30	0.04<br>0.02-0.07	0.03<br>0.02-0.04
IL-19	6.40<br>2.73-8.70	7.43<br>5.16-9.7	7.53<br>4.78-8.52	11.1<br>7.37-21.7	7.04<br>5.77-7.69	7.04<br>6.09-8.52
IL-20	>5810	>5810	>5810	>5810	>5810	>5810
IL-22	5.37<br>1.86-7.86	8.19<br>7.59-8.79	5.98[g]<br>3.78-7.34	12.00[g]<br>7.95-250.00	5.67<br>3.97-5.98	5.67<br>4.59-7.51
IL-26	3.72<br>1.78-4.46	3.77<br>3.49-4.05	4.13<br>3.14-4.91	4.97<br>4.85-20.60	3.57<br>3.18-4.13	4.13<br>3.57-4.46
IL-27	5.64<br>0-13.10	7.48<br>0.79-14.2	8.77<br>0-14.40	20.8<br>8.77-96.4	3.63<br>1.7-12.00	7.71<br>3.14-14.2
IL-28A/IFN-λ2	0<br>0-1813	975<br>0-1951	0[h]<br>0-2219	2508[h]<br>1355-3042	1644<br>0-2070	1065<br>0-2289
IL-29/IFN-λ1	14<br>5.7-22.6	19.3<br>11.5-27.2	17.6<br>12.1-22.5	22.5<br>18.1-53.2	14.8<br>11.5-18.5	14<br>11.5-24.0

IL-32	0	0	0	0	0	0
IL-34	0<br>0-0.5	0.65<br>0-1.31	0.03<br>0-1.85	6<br>0.45-28	0.11<br>0-0.45	0<br>0-0.12
IL-35	0<br>0-7.46	0	0<br>0-5.95	0<br>0-37.4	0<br>0-2.61	0<br>0-1.36
LIGHT/TNFSF 14	3.62<br>0-34.4	5.75<br>0-11.5	0.86<br>0-3.93	3.2<br>1.89-57.2	0<br>0-1.86	0.3<br>0-2.81
MMP-1	28498<br>13517-36965	39611<br>25061-54161	24723<br>19056-31898	28498<br>25045-43179	22641<br>19797-28498	24872<br>20887-29072
MMP-2	1061<br>248-3757	1025<br>794-1256	3176<br>1505-5000	4463<br>2071-6358	3564<br>3396-3771	4720<br>3198-6682
MMP-3	313<br>95-694	566<br>411-720	271<br>121-397	341<br>274-675	286<br>181-608	313<br>215-490
osteocalcin	>208184	>208184	>208184	>208184	>208184	>208184
osteopontin (OPN)	1808<br>458-3181	2660<br>1872-3448	4187<br>1674-5633	4614<br>3011-23151	3157<br>1852-4036	3703<br>2463-5241
pentraxin-3 (PTX-3)	4832<br>4084-6492	5534<br>5235-5833	5776[i]<br>4998-6485	7034[i]<br>6237-8206	6635<br>6204-7213	6156<br>5342-7145
sTNF-R1	398<br>87-870	1348<br>787-1909	543<br>276-804	608<br>334-1148	704<br>381-744	562<br>351-804
sTNF-R2	254<br>0-595	634<br>206-1062	321<br>130-537	437<br>228-688	441<br>389-603	263<br>120-786
TSLP	25.4<br>5.8-36.9	26.4<br>20.3-32.4	24.9[j]<br>19.3-40.7	48.9[j]<br>33.6-65.8	24.1<br>12.6-33.3	25.2<br>18.4-37.4
TWEAK/TNFS F12	56.7<br>9.56-102	122<br>45.3-198	94.8<br>56.4-130	142<br>62.4-167	105<br>74.1-113	76.3<br>48-120

Abbreviations: TB – tuberculosis children; LTBI – children with latent *M.tb* infection, HC – healthy controls; Me – median, IQR – interquartile range.
[a]p=0.02; [b]p=0.04; [c]p=0.0001; [d]p=0.0001;[e]p=0.04; [f]p=0.01; [g]p=0.04; [h]p=0.04; [i]p=0.01; [j]p=0.02.
